# Species that require long-day conditions to flower are not advancing their flowering phenology as fast as species without photoperiod requirements

**DOI:** 10.1093/aob/mcae121

**Published:** 2024-07-31

**Authors:** Karen Zeng, Alexander T Sentinella, Charlotte Armitage, Angela T Moles

**Affiliations:** Evolution & Ecology Research Centre, School of Biological, Earth and Environmental Sciences, UNSW Sydney, NSW 2052, Australia; Evolution & Ecology Research Centre, School of Biological, Earth and Environmental Sciences, UNSW Sydney, NSW 2052, Australia; Woodland Trust, Kempton Way, Grantham, Lincolnshire NG31 6LL, UK; Evolution & Ecology Research Centre, School of Biological, Earth and Environmental Sciences, UNSW Sydney, NSW 2052, Australia

**Keywords:** Climate change, phylogenetic signal, growth form, long day

## Abstract

**Background and Aims:**

Over the last few decades, many plant species have shown changes in phenology, such as the date on which they germinate, bud or flower. However, some species are changing more slowly than others, potentially owing to daylength (photoperiod) requirements.

**Methods:**

We combined data on flowering-time advancement with published records of photoperiod sensitivity to try to predict which species are advancing their flowering time. Data availability limited us to the Northern Hemisphere.

**Key Results:**

Cross-species analyses showed that short-day plants advanced their flowering time by 1.4 days per decade and day-neutral plants by 0.9 days per decade, but long-day plants delayed their flowering by 0.2 days per decade. However, photoperiod-sensitivity status exhibited moderate phylogenetic conservation, and the differences in flowering-time advancement were not significant after phylogeny was accounted for. Both annual and perennial herbs were more likely to have long-day photoperiod cues than woody species, which were more likely to have short-day photoperiod cues.

**Conclusions:**

Short-day plants are keeping up with plants that do not have photoperiod requirements, suggesting that daylength requirements do not hinder changes in phenology. However, long-day plants are not changing their phenology and might risk falling behind as competitors and pollinators adapt to climate change.

## INTRODUCTION

The timing of biological events such as migration, budburst and flowering have shown clear signs of change over recent years (Menzel *et al.*, 2006; Amano *et al.*, 2010). However, these responses are highly variable, with ~70 % of plant species studied advancing their flowering times (Wolkovich *et al.*, 2012; Parmesan and Hanley, 2015; Park and Talbot, 2018). Species that are not able to change their phenology in response to warming are predicted to suffer from missing out on the start of longer growing seasons (Körner and Basler, 2010). Plants responding to warming at different rates might becoming temporally separated from their pollinators, whether pollinators advance faster than plants (Hutchings *et al.*, 2018; Olliff-Yang and Mesler, 2018) or plants advance faster than pollinators [Kudo and Ida, 2013; Stemkovski *et al.*, 2020; but see Freimuth *et al.* (2022) and Bartomeus *et al.* (2011) for evidence that mismatch often does not occur]. Identifying the factors that contribute to how plant taxa change their phenology is thus crucial for conservation efforts and for predicting the likely composition of future ecosystems (Gérard *et al.*, 2020). The main aim of the present study was to determine whether photoperiod sensitivity is associated with reduced changes in flowering time, supporting the idea that photoperiod requirements can limit the ability of species to change their flowering times.

Photoperiod-sensitive plants use daylength to determine the timing of phenological events such as germination and flowering (Garner and Allard, 1920). Well-timed phenology allows plants to take full advantage of favourable growing seasons and allows them to prepare for unfavourable conditions (Hoffmann and Sgrò, 2011). The general mechanism that regulates photoperiod sensitivity involves light-sensitive proteins that interact with vernalization proteins (Yan *et al.*, 2004) and the circadian clock (Johansson and Staiger, 2015) through a complex set of regulatory pathways. After a species-specific set of temperature and day-length requirements are fulfilled, a group of hormones collectively known as florigen are produced, which trigger floral growth (An *et al*., 2004).

By incorporating photoperiod cues into their flowering time, plants lessen their responsiveness to stochastic weather events. This is an advantage when it prevents a species from initiating flowering after a single warm day. However, this same process cannot distinguish between stochastic weather and long-term changes and might therefore be detrimental to a plant under climate change. It is suggested that photoperiod cues might not only hinder adaptive phenological shifts in stationary plant species but might also hinder the ability of plants to shift their ranges latitudinally towards cooler climates (Saikkonen *et al.*, 2012). The theory that sensitivity to photoperiod cues might hold back adaptive phenological shifts was developed with respect to the budburst phenology of deciduous trees (Körner and Basler, 2010; Way and Montgomery, 2015), and evidence for this theory remains based primarily on tree budburst phenology. For example, photoperiod-sensitive deciduous trees have more stable annual budburst timing but experience greater variation in growing degree days than their photoperiod insensitive-counterparts (Zohner *et al.*, 2016). There is, however, no reason to believe that this phenomenon is limited to budburst, with photoperiodic constraints also being hypothesized to influence the phenology of flowering herbs, animal migration and crop harvests (Saikkonen *et al.*, 2012).

If photoperiod sensitivity does hinder flowering-time advance, then knowing which species have photoperiod-sensitive flowering will allow us to predict which species are most likely to fail to respond appropriately to warming climates. However, because measurement of photoperiod sensitivity is labour intensive, data are available for only relatively few species; therefore, we explored whether traits or phylogeny could be used to predict flowering photoperiod sensitivity.

It has been suggested that longer-lived and late successional species are more likely to have photoperiod-sensitivity requirements than opportunistic annual herbs (Körner and Basler, 2010). A previous study (Way and Montgomery, 2015) found that successional status, xylem anatomy or evergreen/deciduous status was not associated with bud burst photoperiod sensitivity in trees. However, there has never been an attempt to determine whether growth form or life history is associated with photoperiod sensitivity in flowering. We tested whether photoperiod sensitivity in flowering is more common in woody and perennial species than in annual herbs.

If photoperiod sensitivity is a strongly conserved trait, it might help to explain phylogenetic trends in phenological advancement identified in other studies (Willis *et al.*, 2008). The possibility that closely related species might share photoperiod sensitivity was proposed >50 years ago (Leopold, 1951), but subsequent studies have since found that photoperiod requirements are not wholly phylogenetically conserved, with cases of highly varied photoperiod requirements being observed between closely related species (Currey and Erwin, 2010) and even within species (Van Dijk and Hautekèete, 2007). We therefore also tested whether there exist patterns of flowering photoperiod sensitivity that have held repeatedly across evolutionary divergences and would be visible as patterns between closely related species.

Our final aim was to determine whether long-day plants and/or short-day plants advance their phenology more slowly than their photoperiod-insensitive counterparts. Slower rates of change in response to altered climatic conditions have been attributed to photoperiod sensitivity in multiple studies (Urbanski *et al.*, 2012; Fu *et al.*, 2015; Jochner and Menzel, 2015; Ford *et al.*, 2017) and one recent review (Renner and Zohner, 2018). It has also been documented that spring leafing in woody plants is less responsive to temperature cues when photoperiod cues are not met (Heide, 1993). However, there have been few attempts to determine the extent to which flowering photoperiod sensitivity affects phenological changes at a large scale. We therefore aimed to find the average rate of flowering-time advancement for long-day plants (requiring >12 h of light and summer flowering), short-day plants (requiring <12 h of light and spring/autumn flowering) and day-neutral plants (photoperiod insensitive) and to determine whether there are substantial differences in flowering-time advancement between the three groups.

In summary, our hypotheses were as follows:

(1) Photoperiod sensitivity will be more common in species with longer lifespans and woody growth.
(2) Photoperiod sensitivity will be phylogenetically conserved.
(3) Long-day species and short-day species will show less flowering-time advancement than day-neutral species.

## MATERIALS AND METHODS

### Photoperiod-sensitivity data

We compiled photoperiod-sensitivity data by searching ISI Web of Science using the keywords ‘flower*’ in combination with the words ‘photoperiod*’ OR ‘long day’ OR ‘short day’ OR ‘neutral day’ in all document types and languages from 1900 to 2020. Exact search terms are available in the Supplementary Data Methods S1. The updated literature search conducted in November 2020 returned 2782 results. We performed a keyword analysis using the ‘revtools’ package (Westgate, 2019) to reduce this to 789 suitable papers (Supplementary Data Methods S2). Additional papers and reference books were added by looking through references that original papers identified in the online search, in addition to highly cited literature reviews. Sources for data are available in the Supplementary Data Methods S3.

Each paper was checked for experimental data relating to photoperiod-sensitivity cues. Floral photoperiod experiments are generally performed by treating plants with a continuous scale of daylengths. The detection of a photoperiod cue is observed as variations in the amount of flowering initiated under each treatment daylength, with the treatment that produces the most flowering determining the category of the photoperiod cue.

We recorded the type of photoperiod sensitivity exhibited and the data source for each species as described in the source. We attempted to obtain all primary sources but were unable to access a few old texts, and therefore had to rely on the accuracy of secondary sources in rare cases. Data for agricultural crops were excluded from the study because they are likely to be subject to selective breeding towards artificial flowering-time responses (Nakamichi, 2015). We also excluded species that do not have an annual flowering season, owing to the difficulties of quantifying phenological advance without consistent phenology. In cases of conflicting results, we preferred records that did not use cultivars, methods that tested more daylength intervals and experiments conducted more recently (because light quality was an issue in early experiments). We then categorized the wide variety of photoperiod responses into one of three categories of photoperiod sensitivity: long day, short day and day neutral. We define long-day and short-day plants as those with maximum flowering under daylengths >12 and <12 h, respectively, and day-neutral plants as those whose flowering rates are unaffected by daylength. We did not consider whether photoperiod cues were obligate or facultative, because this was often not tested in the primary literature. Species with complex flowering requirements that could not be assigned to long-day, short-day or day-neutral categories were excluded from the analysis. When a species showed differing photoperiod responses based on temperature, we selected the photoperiodic response at the higher temperature owing to its relevance regarding climate change. For example, Heide (2002) found that species of *Carex* generally have short-day photoperiod cues but move increasingly towards day-neutral and even long-day requirements in low-temperature, high-elevation populations. This search resulted in photoperiod-sensitivity data for 767 species spanning 91 sources (Supplementary Data Table S1). Of these species, 569 were considered photoperiod sensitive in some way.

### Life-history and growth-form data

We obtained life-history data (annual/perennial) from the TRY plant database (Kattge *et al.*, 2011). We also adapted woodiness data (woody/herbaceous) from Zanne *et al.* (2009), under the assumption that woody species were perennial. Species that could not be classified readily into these groups were excluded from the relevant analyses. By combining the two, we were able to categorize 328 species as perennial woody, perennial herb or annual herb species.

### First flowering day

From existing publicly available datasets we extracted the first flowering date, a standard measure of plant phenology with evolutionary importance and an abundance of data. Although other measurements of flowering, such as peak flowering time and flowering distribution, are less susceptible to changes in population size and sampling frequency (Miller-Rushing and Primack, 2008), first flowering date remains the most widely used measurement of flowering time, with historical datasets being freely available and constantly updated (Willis *et al.*, 2008; Primack *et al.*, 2009; Amano *et al.*, 2010).

Flowering-time data were collated from a combination of published papers and phenological networks (Supplementary Data Table S2). Global compilations suggest an average phenological advancement of between 2.3 and 5.1 days per decade (Parmesan and Yohe, 2003; Root *et al.*, 2003). Therefore, we did not consider individual studies that ran for a total of <5 years or that sampled at intervals longer than once every 5 days, because they are unlikely to identify changes in phenology. We considered four previous compilations of flowering-time data that met our criteria (Abu-Asab *et al.*, 2001; Fitter and Fitter, 2002; Davis and Ellison, 2015; Park *et al.*, 2019). We complemented the data from published literature with data provided by four phenological networks: the Pan European Phenology Project (Templ *et al.*, 2018), the Rocky Mountain Biological Laboratory Phenology Project, Nature’s Calendar (Woodland Trust, UK) and the USA National Phenology Network (Rosemartin *et al.*, 2015). Where coordinates were otherwise unavailable, we obtained them from Google Earth. We updated taxonomic nomenclature and removed duplicates under different species names using information on The Plant List (http://www.theplantlist.org/).

**Fig. 1.**
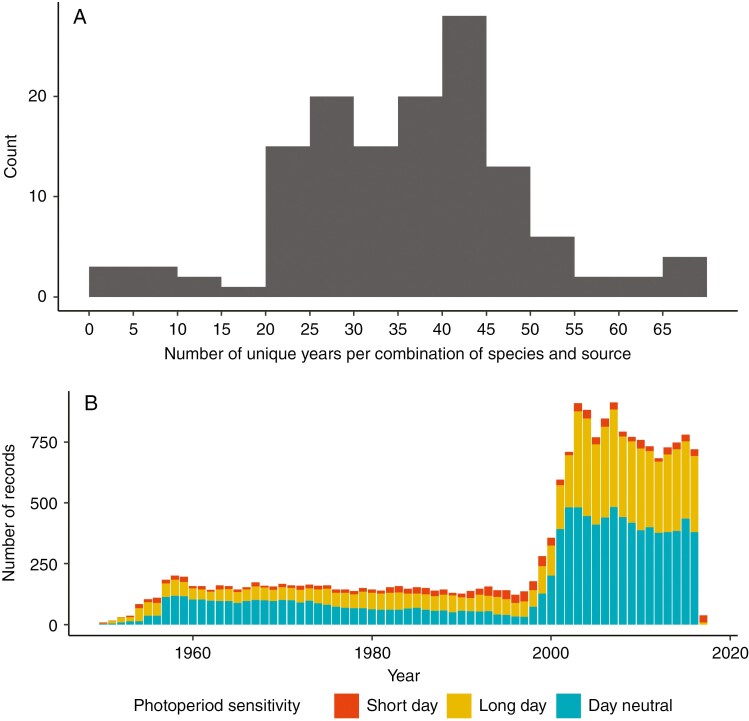
Distribution of combined photoperiod and first flowering day data. (A) The number of unique years of data occurring per species per source. (B) The distribution of first flowering dates with matching photoperiod-sensitivity information across time.

We took the average of the first flowering date for each year per species per source for each location determined by rounding to the nearest full degree of latitude and longitude (Fig. 1). By grouping together very similar records, we were able to balance the weight of citizen science data (high variability but many samples) with peer-reviewed experiments (lower variability but fewer samples). We also added a correction to account for leap years by isolating the day of year measurements using the ‘lubridate’ package (Grolemund and Wickham, 2011), then multiplying each of these values by 365/366 to ensure that they represented the same proportion of the year as non-leap-year values. Where possible, we converted available coordinates into WGS84; otherwise, approximate coordinates were obtained using either Google Earth or ‘rgeos’ (Bivand *et al.*, 2017) to identify the centroid of a named location field (in the form of country, county or field station). This spatial data manipulation was performed in R using the ‘rgdal’ (Bivand *et al.*, 2015), ‘rworldmap’ (South, 2011) and ‘sp’ (Pebesma and Bivand, 2005) packages.

### Distribution of photoperiod-sensitivity and flowering-time data

**Fig. 2.**
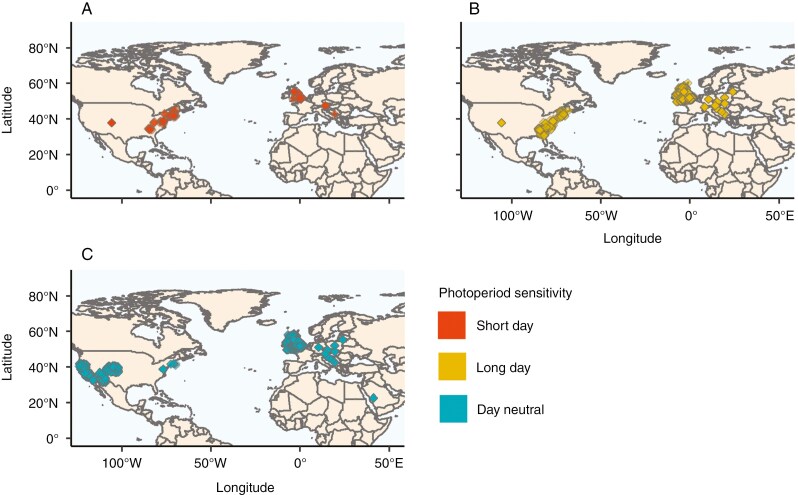
Geographical distribution of combined photoperiod and first flowering day data. Geographical distribution of first flowering dates with associated photoperiod sensitivity for short-day (A), long-day (B) and day-neutral (C) species.

All records with complete data for both photoperiod sensitivity and flowering time were from the Northern Hemisphere (Fig. 2). Two implications of this fact are: (1) there is a striking knowledge gap around flowering-time advances in the Southern Hemisphere; and (2) we did not need to account for hemisphere when analysing our flowering-time data or deal with species whose flowering time spanned the start and end of the year.

We were able to obtain sufficient flowering-time data for 88 species for which we had photoperiod-sensitivity information (additional information is available in Supplementary Data Methods S4). Of these 88 species, none was noted as a cultivar. This was reduced further, to 62 species, in our phylogenetic analysis, owing to the per-species analysis requiring multiple records per species per location, in addition to a match on the phylogenetic tree.

### Data analysis

Statistical analyses were performed using R v.3.4.1 (R Core Team, 2022), and data were manipulated using the ‘tidyverse’ package (Wickham *et al.*, 2019). Graphs were produced using ‘ggplot2’ (Wickham, 2016), and ‘ggtree’ and ‘phylobase’ were used to visualize the phylogenetic tree (Yu *et al.*, 2017; Bolker *et al.*, 2020). Exact analyses are available on GitHub (https://github.com/karenzeng95/Photoperiod-sensitive-plants-have-lower-rates-of-flowering-time-advancement); tables of outputs are available in Supplementary Data Methods S5, and list of species used in each analysis is available in Supplementary Data Methods S6.

### What type of species are most likely to have flowering photoperiod sensitivity?

We performed χ^2^ tests comparing photoperiod sensitivity and its groups with growth form (woody plant, perennial herb or annual herb) using the ‘chisq.test’ command from base R.

### Does photoperiod sensitivity exhibit a phylogenetic signal?

Phylogenetic relationships were obtained by pruning the phylogenetic tree published by Smith and Brown (2018) using the ‘ape’ package (Paradis *et al.*, 2004). Multichotomies in the tree were resolved by transforming branch lengths of zero to a number less than half of the smallest existing branch length (0.001). This pruned tree included a total of 476 species.

The phylogenetic signal of the categorical photoperiod-sensitivity types was calculated by comparing our delta statistic with those of partly and fully randomized comparison datasets as described by Borges *et al.* (2019).

### Change in first flowering day over time and its interaction with photoperiod sensitivity

We fitted a generalized linear mixed model between first flowering day and year of observation to determine the change in first flowering day over time.


$$\begin{array}{l} First\;flowering\;day∼year+latitude+\left(1|species\right) \end{array}$$

Each replicate in this analysis was the first flowering day per year per source for one species within one degree of latitude and longitude. We included a fixed effect for latitude as a strong predictor of within-species variation (Debieu *et al.*, 2013), capturing information about seasonal fluctuations in daylength and climate. Flowering time differs between species (Parmesan and Yohe, 2003; Root *et al.*, 2003); therefore, we also we included a random effect for species in our analysis.

We assessed whether photoperiod sensitivity was correlated with changes in first flowering day throughout the years by comparing the model above with a model that included a term for how different photoperiod types change phenology over time (photoperiod sensitivity:year). Latitude interacts directly with photoperiod cues via its control of daylength (Garner and Allard, 1920); therefore, we included latitude as an interaction term with photoperiod sensitivity.


$$\begin{array}{llll} & First\;flowering\;day∼year+photoperiod\;sensitivity\; & +photoperiod\;sensitivity:year\; & +photoperiod\;sensitivity:latitude+\left(1|species\right) \end{array}$$

Generalized linear mixed models were produced using the *lmer* function in the ‘lme4’ package in R (Bates *et al.*, 2015) with the ‘lmerTest’ package to obtain *P*-values using Satterwaithe’s method of estimating degrees of freedom (Kuznetsova *et al.*, 2017).

### Do long-day or short-day plants differ from day-neutral plants in their change in flowering time?

We determined whether the change in flowering time of the three photoperiod categories differed from one another by producing estimated marginal trends through time (including 95 % confidence intervals) for each photoperiod category and contrasts between each trend using *emtrends* from the ‘emmeans’ package (Lenth, 2022). The intercepts and slopes from the model on which we based the trends were then used to calculate average first flowering dates for each photoperiod category across the time frame of our records (1950–2017) to explain our findings better, given that estimated trends themselves provide no intercept on which to base the flowering times.

After finding evidence of a moderate phylogenetic signal in our photoperiod-sensitivity dataset, we decided to perform an additional analysis of flowering-time advancement that included a term for phylogenetic relationships between the species in our dataset.

Our phylogenetic analyses can include only one value per species; therefore, instead of repeating the above analysis for phylogeny, we analysed the relationship between rate of change and photoperiod requirements for each species. We calculated an average change in flowering time per year for each species using a linear mixed-effects model incorporating latitude as a random effect. We then used the average yearly change in flowering time for each species to run a phylogenetic ANOVA between the change through time of each species, their photoperiod requirements and the phylogeny of the species using *phylANOVA* from the ‘phytools’ package (Revell, 2012).

## RESULTS

**Fig. 3.**
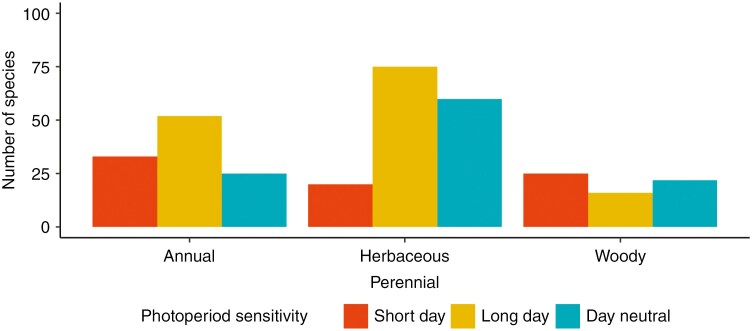
Proportion of photoperiod sensitivity by life history and growth form. Boxplot of proportion of photoperiod sensitivity within herbaceous annuals (*n* = 110), herbaceous perennials (*n* = 155) and woody perennials (*n* = 63).

The occurrence of photoperiod sensitivity in flowering time was significantly associated with growth form (χ^2^ = 8.63, d.f. = 2, *P *< 0.05; Fig. 3). Herbaceous annual plants most often exhibited photoperiod requirements (77.3 % of 110 species), followed by woody plants (65.1 % of 63 species) and herbaceous perennials (61.4 % 125 species). We also found differences in the type of photoperiod sensitivity between growth forms (χ^2^ = 27.31, d.f. = 4, *P *< 0.001). Herbaceous annual species were slightly more likely to require long days to flower (47.3 %) than short days (30 %). Herbaceous perennials were more often long day (48.4 %) than short day (13 %). Woody plants were, in contrast, less often long day (25.4 %) and more often short day (39.7 %), but by only a small margin.

**Fig. 4.**
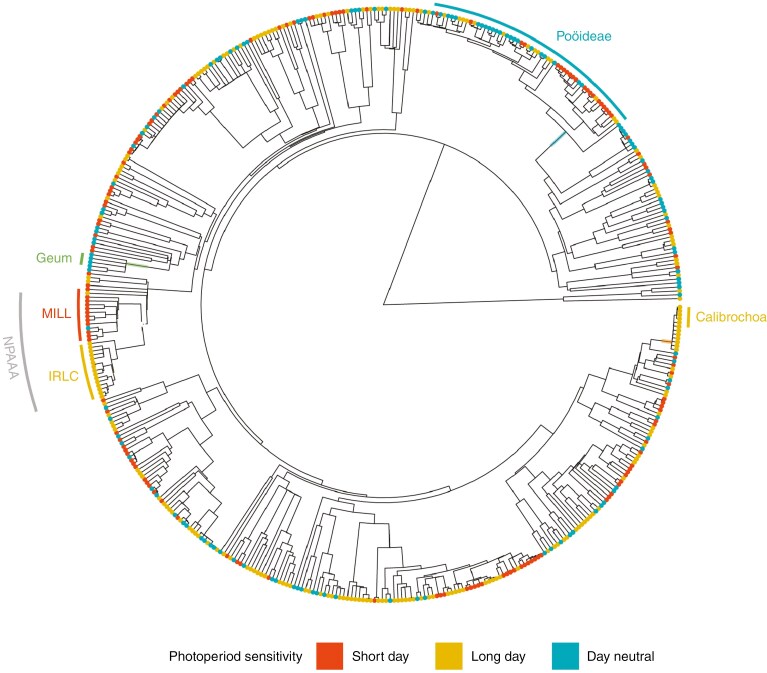
Photoperiod-sensitivity data overlaid onto a phylogenetic tree (*n = *476 species), with identified trends highlighted within Pooideae, *Calibrochoa*, *Geum* and the non-protein amino acid-accumulating clade (NPAAA) containing the core millettioid clade (MILL) and inverted repeat-lacking clade (IRLC).

Our dataset included 667 species from 389 genera and 92 families. We had fewer Orchidaceae (2 species) and more Asteraceae (92 species) in our dataset than would have been expected based on the world’s flora (Lughadha *et al.*, 2016). Photoperiod-sensitivity type exhibited a moderate phylogenetic signal (δ = 1.63, in comparison to δ = 0.15 for a fully randomized set of data). Visual exploration of the data (Fig. 4) revealed that all 12 species of *Calibrochoa* in our dataset had long-day flowering, all five species of *Geum* were day neutral, and there was an unusually high proportion of day-neutral species within Pooideae. Apart from *Phaseolus vulgaris*, all species in the non-protein amino acid-accumulating (NPAAA) clade (within the subfamily *Faboideae*) were photoperiod sensitive. We also found evidence that a divergence between two major groups within the NPAAA clade resulted in the preference for different types of photoperiod sensitivity, with the core millettioid clade being short day (MILL, a group including *Glycine*, *Phaseolus* and *Desmodium*), whereas the inverted repeat-lacking clade (IRLC, including *Pisum*, *Trigonella* and *Trifolium*) was almost entirely long day. Although there were some phylogenetic trends, we do not expect the taxonomic spread of our dataset to have affected our findings substantially, because photoperiodism is only moderately conserved and our dataset is taxonomically diverse.

We determined that the photoperiod information improved the fit of the model (and would therefore justify further analysis) by comparing the Akaike information criteria of the two models. We confirmed with a type 2 ANOVA between the two models that photoperiod-sensitivity category did have an impact on flowering time (*P* < 0.0001).

**Fig. 5.**
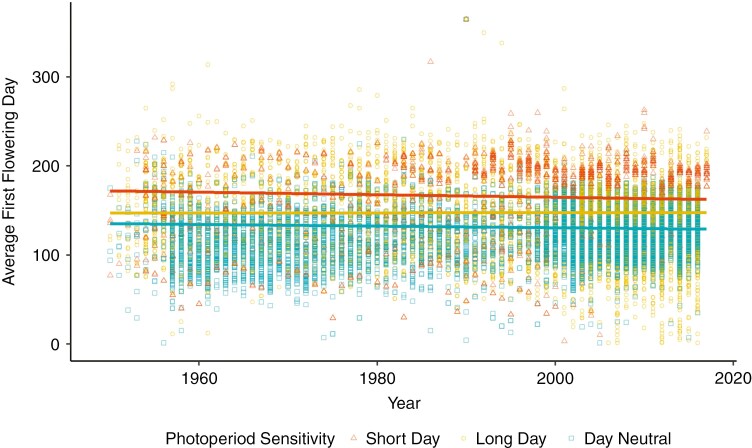
Flowering time advancement. First flowering day for short-day plants (*n =* 1396 records, 28 species), long-day plants (*n =* 9815 records, 35 species) and day-neutral plants (*n =* 10 402 records, 25 species) with trend lines from our generalized linear model (assuming average latitude for each group). Plants are defined as short day if flowering is promoted by daylengths of <12 h (common in spring/autumn-flowering plants), long day if flowering is promoted by daylengths of >12 h (summer flowering) and day neutral if no specific daylength was found to promote flowering.

Based on the second model, the effect of photoperiod-sensitivity type (long-day, short-day and day-neutral species) on flowering-time change was not large (*n* = 21 613 records, *R*^2^ = 0.0236037), but explained significant variation according to an ANOVA (*P* = 0.02). In 1950, the average day-neutral plant began flowering on 15 May, the average short-day plant on 21 June and the average long-day plant on 27 May (Fig. 5). In 2017, the average day-neutral plant began flowering on 9 May, the average short-day plant on 12 June and the average long-day plant on 28 May. Hence, short-day plants advanced their flowering time by 1.42 days per decade, a rate not substantially different from that of day-neutral plants (*z *= 1.005, *P* = 0.57), which advanced by 0.94 days per decade. The rate of change in long-day plants was 0.18 days later per decade, (standard error = 0.19) significantly different from the rate of change in both short-day plants (*z *= 3.343, *P* = 0.002) and day-neutral plants (*z *= −4.276, *P* < 0.001). Cross-species analysis thus showed that short-day plants and day-neutral plants were advancing their flowering time substantially more than long-day plants, and long-day plants were changing at a rate not significantly different from zero [95 % confidence interval (−0.018, 0.054)].

Phylogenetic analysis showed no significant difference in flowering-time change between long-day, short-day and day-neutral species after accounting for phylogenetic signal (*F* = 3.925, *P* = 0.08). This analysis was necessarily performed on a subset of the data; therefore, we ran a cross-species analysis (i.e. not accounting for phylogeny) using the same dataset. This analysis yielded a significant correlation (*F* = 3.93, *P* = 0.03), suggesting that the difference between the phylogenetic analysis and the cross-species analysis on the full dataset was caused by accounting for phylogeny rather than sample size. Within the subset of species for which we ran individual linear models, we found the greatest significant advancement in *Helianthus annuus* at an outlying (and unsustainable) 68 days per decade, followed by *Vaccinium angustifolium* at 10 days per decade and *Vaccinium corymbosum* at 9 days per decade. *Cynosurus cristatus* showed the greatest delay in flowering time, at 0.02 days per decade.

## DISCUSSION

By analysing how plants with different photoperiod requirements have shifted their flowering phenology over the past 67 years, we have found that plants requiring short days to flower were, on average, able to keep up with plants without photoperiod requirements, but plants that require long days to flower were, on average, not changing substantially over time (Fig. 5). Long-day plants might therefore be disadvantaged as they miss out on extended growing seasons (Körner and Basler, 2010) or the potential to become more synchronized with their pollinators (Freimuth *et al.*, 2022). On the contrary, short-day species that change their phenology too rapidly might experience an increased incidence of frost damage (Inouye, 2008; Thomson, 2010; Augspurger, 2013; Ma *et al.*, 2019).

Our finding that plants requiring long days to flower are not changing their flowering time as quickly as short-day or day-neutral plants (Fig. 5) might be explained, in part, by an interaction between photoperiod sensitivity and temperature in shaping flowering time. For day-neutral species, temperature is likely to be the most important cue for flowering (Yan *et al.*, 2004). Short-day plants are also likely to have strong temperature cues for flowering in addition to photoperiod sensitivity to distinguish between spring and autumn occurrences of their ideal daylength. In contrast, long-day plants flower when temperatures are high; hence, they are less likely to have temperature requirements and less likely to change their flowering times in response to climate change. Consistent with this idea, previous work has shown that early-flowering species have advanced their flowering phenology more rapidly than later-flowering species (Price and Waser, 1998; Fitter and Fitter, 2002; Moore and Lauenroth, 2017) and that the flowering time of early-flowering species is more closely correlated with changes in temperature than is the flowering time of later-flowering species (Miller-Rushing and Primack, 2008). A worthwhile direction for future research will be to perform studies that explore further the interacting effects of temperature and daylength in shaping flowering-time change.

Phylogenetic and cross-species analyses are complementary (de Bello *et al.*, 2015). Cross-species analyses tell us whether there is a present-day relationship between two variables, whereas phylogenetic analyses ask whether the relationship between variables has been consistent throughout evolutionary history (Westoby *et al.*, 1995). Our initial questions were at a cross-species level; hence, the results of the cross-species analyses are the focus of most of our discussion and interpretation (following de Bello *et al.*, 2015). However, in asking whether photoperiod sensitivity was more similar in closely related species, we discovered significant phylogenetic signal. We next checked to see whether the difference in flowering-time change between long-day, short-day and day-neutral species remained significant after accounting for phylogeny and found that it did not. The difference between the cross-species results and the phylogenetic results suggests that the relationship between photoperiod and flowering-time change is underpinned by one or more deep divergences in the evolutionary history of plants. That is, the association between photoperiod sensitivity and flowering-time change has not held consistently throughout the evolutionary history of the species in our study. Understanding how this relationship arose is important, but it remains the case that there is an association between photoperiod sensitivity and flowering-time advancement across present-day species.

Short-day and day-neutral plants advanced at 1.4 and 0.9 days per decade, respectively. The current estimated average rate of phenological advancement across all forms of terrestrial life is between 2.3 and 5.1 days per decade globally (Parmesan and Yohe, 2003; Root *et al.*, 2003), with regional estimates and those based on herbaria and citizen science tending to be more variable but at similar scales (Amano *et al.*, 2010; Calinger *et al.*, 2013; Liu *et al.*, 2016; Park *et al.*, 2019; Menzel *et al.*, 2020; Hassan *et al.*, 2023), and plants (especially herbs and shrubs) making up the lower end of the spectrum at 1.1 days per decade (Parmesan, 2007). Although the rates of change observed in our study are relatively small, there is evidence that small shifts in flowering phenology can result in biologically substantial effects over time. For example, a delay of 10 days over 300 years (0.33 days per decade) was found to disrupt the pollination strategy of the sexually deceptive orchid *Ophrys sphegodes* (Hutchings *et al.*, 2018).

We found that flowering photoperiod sensitivity is most common in annual herbs, contrary to the suggestion that photoperiod sensitivity is more common in late-successional species (Körner and Basler, 2010). Instead, photoperiod requirements in flowering were common amongst all growth forms, being most common in annual herbs (Fig. 3). Differences between growth forms instead occurred in the type of daylength required for flowering. Woody species tended to have short-day flowering cues, whereas annual and perennial herbs more often required long days to flower. The higher proportion of short-day flowering in woody species might be because they are more resilient against the increased risk of frost damage experienced by early-flowering species (Ma *et al.*, 2019). That is, a tree that suffers frost damage during flowering is protected by its woody growth and has more chances to flower again and recoup its losses. If a herb mistimes its flowering, it might die and leave no offspring at all. Although annual herbs lose their single chance of reproducing, perennial herbs risk the most by losing both their current and future chances to flower. The increased proportion of long-day photoperiod cues in herbaceous species might thus be a safer strategy, given that flowering in the peak of summer avoids potential frost damage at the cost of a more limited growing window.

Given the role of photoperiod sensitivity in the ability of species to respond to climate change, it is concerning how little information exists on the photoperiod requirements that control the many stages of phenology (germination, flowering, budburst and tuberization). Furthermore, much of the primary literature that tests for photoperiod sensitivity is from the early 20^th^ century, when antiquated lighting and temperature controls often precluded the accuracy expected in modern testing conditions. We also found that although photoperiod-sensitivity cues are more likely in certain growth forms and might be somewhat conserved within clades, these associations cannot reliably predict the occurrence of flowering photoperiod cues. Thus, we are still facing a shortage of data on flowering photoperiod sensitivity for species other than crops. Our search for data also uncovered a gap in long-term data for Southern Hemisphere species. Although we did find information on the photoperiod cues of a few Southern Hemisphere plants (King, 1998; Shen *et al.*, 2021), we were unable to match a single long-term dataset on flowering time from the Southern Hemisphere with a recorded photoperiod sensitivity, limiting our study to the Northern Hemisphere. However, with improved growing techniques and promising advances in identifying molecular timers that can be used to predict photoperiod sensitivity (Gao *et al.*, 2019), we are now able to collect data on photoperiod requirements more accurately than ever. The emergence of large-scale databases of plant traits (Kattge *et al.*, 2011) and citizen science initiatives (Callaghan *et al.*, 2021) has also laid the groundwork in allowing for the collection and dissemination of photoperiod requirements in a standardized way. Although photoperiod is only one piece of the puzzle of phenology, it is a promising avenue for further research that can not only benefit fundamental ecological knowledge, but also lead to benefits for conservation and climate modelling.

## SUPPLEMENTARY DATA

Supplementary data are available at *Annals of Botany* online and consist of the following.

Supplementary Data Methods S1 to S6: search terms used in study, search methodology, source data references, README text and descriptions, summary of model outputs and list of species used in each analysis. Supplementary Data Table S1: photoperiod data. Supplementary Data Table S2: flowering time data.

mcae121_suppl_Supplementary_Materials

## Data Availability

Additional analysis code is available at https://github.com/karenzeng95/Photoperiod-sensitive-plants-have-lower-rates-of-flowering-time-advancement.
