# Supplementary material for: Species that require long-day conditions to flower are not advancing their flowering phenology as fast as species without photoperiod requirements
Source: Ann Bot. 2024 Jul 31;135(1-2):113–24. doi: 10.1093/aob/mcae121 (PMC11979757; doi:10.1093/aob/mcae121)
Supplement: mcae121_suppl_Supplementary_Materials [file mcae121_suppl_supplementary_materials.zip › mcae121_Supplementary_methods_data_S1-6.docx]

**Supporting Information for study titled: Species that require long day conditions to flower are not advancing their flowering phenology as fast as species without photoperiod requirements.**

Authors: Karen Zeng^1*^, Alexander T. Sentinella^1^, Charlotte Armitage^2^, Angela T. Moles^1^

The following Supporting Information is available in this document:

**Supplementary methods 1:** Search terms used in study

**Supplementary methods 2:** Revtools search methodology

**Supplementary methods 3:** Source data references

**Supplementary methods 4:** README text and descriptions for supplementary tables.

**Supplementary methods 5:** Summary of model outputs

**Supplementary methods 6:** List of species used in each analysis

**Methods S1** Search terms used in study

Photoperiod data were obtained using ISI Web of Science in August 2017, and then updated in November 2020 using the same search terms:

TS = ("flower*" AND ("photoperio* sensitivity" OR "photoperiodism" OR "long day" OR "short day" OR "neutral day")) NOT TS = ("arabidopsis" OR "maize" OR "triticum" OR "glycine" OR "strawberry" OR "duckweed" OR "lemna" OR "crop" OR "crops" OR "gibbere*" OR "cytokin*")

TOPIC: (phenology) OR TOPIC: (flowering time)

Refined By: DOCUMENT TYPES: (ARTICLE) AND DOCUMENT TYPES: (ARTICLE) AND [excluding]: WEB OF SCIENCE CATEGORIES: (CHEMISTRY MEDICINAL OR AGRONOMY OR OCEANOGRAPHY OR ENTOMOLOGY OR HORTICULTURE OR BEHAVIORAL SCIENCES OR AGRICULTURE DAIRY ANIMAL SCIENCE OR DEVELOPMENTAL BIOLOGY OR ZOOLOGY OR BIOTECHNOLOGY APPLIED MICROBIOLOGY OR VETERINARY SCIENCES OR MARINE FRESHWATER BIOLOGY OR FOOD SCIENCE TECHNOLOGY OR IMAGING SCIENCE PHOTOGRAPHIC TECHNOLOGY OR FISHERIES OR PHARMACOLOGY PHARMACY OR CHEMISTRY ANALYTICAL) AND DOCUMENT TYPES: (ARTICLE) AND [excluding]: WEB OF SCIENCE CATEGORIES: (GENETICS HEREDITY OR AGRICULTURE MULTIDISCIPLINARY) AND [excluding]: WEB OF SCIENCE CATEGORIES: (METALLURGY METALLURGICAL ENGINEERING OR ENERGY FUELS OR CHEMISTRY INORGANIC NUCLEAR OR PUBLIC ENVIRONMENTAL OCCUPATIONAL HEALTH OR ENGINEERING ELECTRICAL ELECTRONIC OR MATHEMATICAL COMPUTATIONAL BIOLOGY OR POLYMER SCIENCE OR AGRICULTURAL ENGINEERING OR ELECTROCHEMISTRY OR ENGINEERING CIVIL OR CHEMISTRY PHYSICAL OR MATERIALS SCIENCE CERAMICS OR PHYSICS APPLIED OR TOXICOLOGY OR PSYCHOLOGY EXPERIMENTAL OR URBAN STUDIES OR PHYSICS CONDENSED MATTER OR NEUROSCIENCES OR NANOSCIENCE NANOTECHNOLOGY OR ANTHROPOLOGY OR ENGINEERING CHEMICAL OR BIOCHEMICAL RESEARCH METHODS OR CRYSTALLOGRAPHY OR MICROBIOLOGY OR MATERIALS SCIENCE COATINGS FILMS OR PARASITOLOGY OR MYCOLOGY) AND [excluding]: DOCUMENT TYPES: (BOOK CHAPTER OR RETRACTED PUBLICATION).

**Methods S2** Revtools search methodology

We used ‘revtools’ to identify the most common topics that appeared in the initial search, as well as the keywords which defined the topics covered. This helped us pick specific words to include or exclude to catch all the photoperiod sensitivity data while removing as much irrelevant literature as possible. For example, we identified that genetic regulation made up a large proportion of papers but did not contribute to photoperiod data, so we excluded terms such as ‘arabidopsis’, ‘transcription’ and ‘cytokin*’.

Below are the outputs for the various settings used:

Appendix Table 1: Revtools outputs

| Model Type = CTM  Iterations = 2000  Topics = 4  Topic #1  Top terms: flowering, genes, expression, plants, arabidopsis  Other relevant terms: regulation, protein, transcription, cell, mutant  Topic #2  Top terms: flowering, species, seed, plants, pollination  Other relevant terms: fruit, pollen, reproductive, populations, female  Topic #3  Top terms: phenology, season, species, change, climate  Other relevant terms: temperature, forest, years, trees, spring  Topic #4  Top terms: plants, using, growth, time, effects  Other relevant terms: crop, methods, concentration, yield, root | 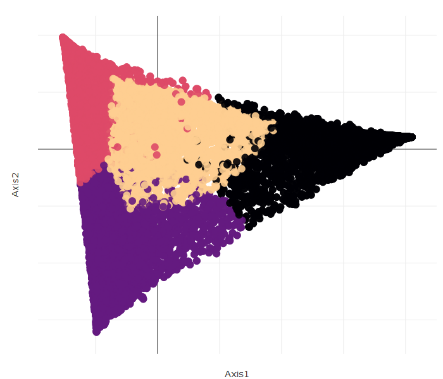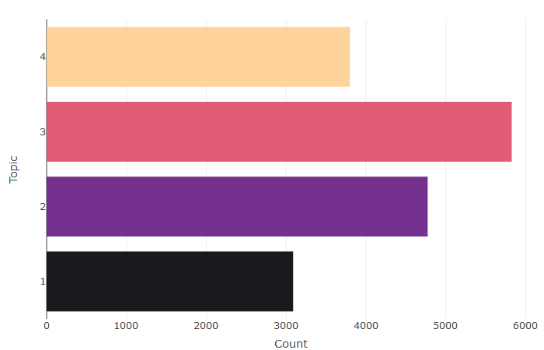 |
| --- | --- |
| Model Type = LDA  Iterations = 2000  Topics = 4  Topic #1  Top terms: phenology, change, using, temperature, climate  Other relevant terms: model, data, vegetation, region, spring  Topic #2  Top terms: growth, plants, effects, increased, leaf  Other relevant terms: trees, production, forest, season, soil  Topic #3  Top terms: flowering, genes, plants, time, development  Other relevant terms: expression, activity, function, arabidopsis, regulation  Topic #4  Top terms: species, flowering, populations, pollination, fruit  Other relevant terms: reproductive, pollen, size, seed, individuals | 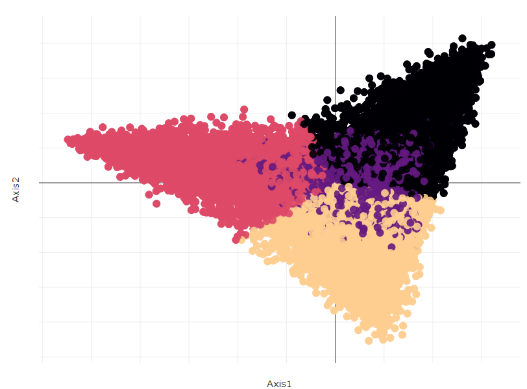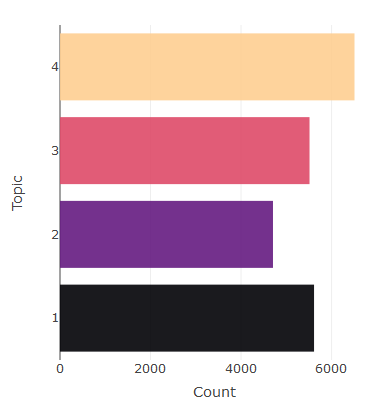 |
| Model Type = LDA  Iterations = 2000  Topics = 5  Topic #1  Top terms: flowering, plants, seed, populations, pollination  Other relevant terms: fruit, reproductive, pollen, size, individuals  Topic #2  Top terms: growth, leaf, trees, forest, season  Other relevant terms: soil, water, increased, leaves, dry  Topic #3  Top terms: species, study, different, traits, two  Other relevant terms: distribution, new, structure, community, morphology  Topic #4  Top terms: phenology, change, temperature, climate, model  Other relevant terms: data, vegetation, using, spring, date  Topic #5  Top terms: flowering, genes, plants, expression, development  Other relevant terms: arabidopsis, function, activity, regulation, protein | 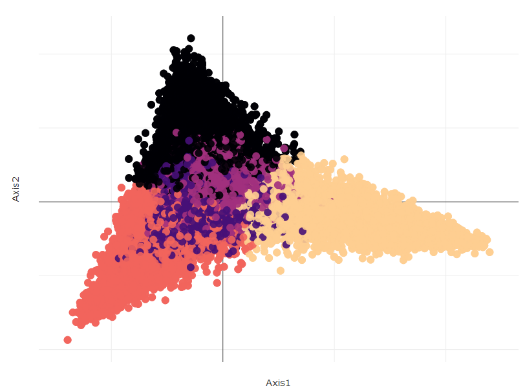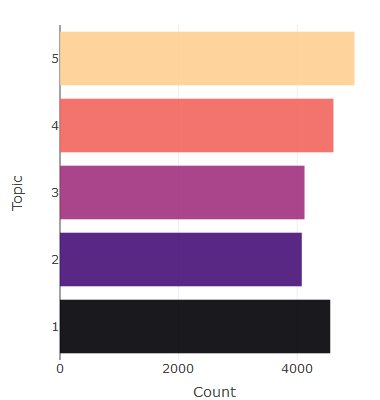 |

**Methods S3** Source data references

**Adams SR, Pearson S, Hadley P**. **1997**. The Effects of Temperature, Photoperiod and Light Integral on the Time to Flowering of Pansy cv. Universal Violet (Viola×wittrockiana Gams.). *Annals of Botany* **80**: 107–112.

**Armitage AM**. **1995**. Photoperiod, Irradiance, and Temperature Influence Flowering of Hamelia patens (Texas Firebush). *HortScience* **30**: 255–256.

**Armitage AM, Garner JM**. **1999**. Photoperiod and cooling duration influence growth and flowering of six herbaceous perennials. *The Journal of Horticultural Science and Biotechnology* **74**: 170–174.

**Baloch J-D, Munir M, Abid M**. **2013**. Flowering response of facultative short day ornamental annuals to artificial light intensities. *Pak. J. Bot* **45**: 999–1004.

**Baskin JM, Baskin CC**. **1998**. Greenhouse and Laboratory Studies on the Ecological Life Cycle of Dalea foliosa (Fabaceae), a Federal Endangered Species. *Natural Areas Journal* **18**: 54–62.

**Bassett CL, Mothershed CP, Galau GA**. **1991**. Polypeptide profiles from cotyledons of developing and photoperiodically induced seedlings of the Japanese morning glory (Pharbitis [ipomoea] nil). *Journal of Plant Growth Regulation* **10**: 147.

**Bhargava SC**. **1965**. Inhibition of Flowering by Light in the Short-Day Plant Salvia occidentalis. *Science*

**147**: 60–61.

**Boyle T.h**. **1991**. Temperature and photoperiodic regulation of flowering in ‘Crimson Giant’ Easter cactus. *Journal of the American Society for Horticultural Science*.

**Castro JC, Boe A, Lee DK**. **2011**. A Simple System for Promoting Flowering of Upland Switchgrass in the Greenhouse. *Crop Science* **51**: 2607–2614.

**Chouard P**. **1946**. Sur le photopériodisme chez les plantes vivaces. *Bulletin de la Société Botanique de France* **93**: 373–377.

**Chouard P**. **1960**. Vernalization and its Relations to Dormancy. *Annual Review of Plant Physiology* **11**: 191–238.

**Cleland CF, Briggs WR**. **1967**. Flowering Responses of the Long-day Plant Lemna gibba G3 1. *Plant Physiology* **42**: 1553–1561.

**Cooper JP, Calder DM**. **1964**. The Inductive Requirements for Flowering of Some Temperate Grasses.

*Grass and Forage Science* **19**: 6–14.

**Criley RA, Sakai WS**. **1997**. Heliconia wagneriana Petersen is a Short-day Plant. *HortScience* **32**: 1044–1045.

**Currey C, Erwin J**. **2010**. Variation among Kalanchoe species in their flowering responses to photoperiod and short-day cycle number. *The Journal of Horticultural Science and Biotechnology* **85**: 350–354.

**Damann MP, Lyons RE**. **1993**. Juvenility, Flowering, and the Effects of a Limited Inductive Photoperiod in Coreopsis grandiflora and C. lanceolata. *Journal of the American Society for Horticultural Science* **118**:

513–518.

**Friedman J, Willis JH**. **2013**. Major QTLs for critical photoperiod and vernalization underlie extensive variation in flowering in the Mimulus guttatus species complex. *New Phytologist* **199**: 571–583.

**Garner W, Allard H**. **1923**. *Further studies in photoperiodism: the response of the plant to relative length of day and night*. US Government Printing Office.

**Guo Z, Goi M, Tanaka M, Fukai S**. **1993**. Effects of Temperature and Photoperiod on the Bud Formation of Camellia wabisuke’Tosauraku’. *Journal of the Japanese Society for Horticultural Science* **61**: 911–918.

**Gutterman Y**. **1997**. Effect of daylength on flowering and seed morphology ofSpergularia diandraoccurring in the Negev Desert, Israel. *Journal of Arid Environments* **36**: 611–622.

**Han T, Xu E, Yao L, Zheng B, Younis A, Shao Q**. **2020**. Regulation of flowering time using temperature, photoperiod and spermidine treatments in Anoectochilus roxburghii. *Physiology and Molecular Biology of Plants* **26**: 247–260.

**Heide OM**. **1992**. Flowering strategies of the high-arctic and high-alpine snow bed grass species Phippsia algida. *Physiologia Plantarum* **85**: 606–610.

**Heide OM**. **1994**. Control of flowering and reproduction in temperate grasses. *New Phytologist* **128**: 347–362.

**Heide OM**. **2002**. Climatic flowering requirements of bipolar sedges, Carex spp., and the feasibility of their trans-equatorial migration by mountain-hopping. *Oikos* **99**: 352–362.

**Heide OM**. **2004**. Dual induction rather than intermediate daylength response of flowering in Echinacea purpurea. *Physiologia Plantarum* **120**: 298–302.

**Heide OM**. **2005**. Ecotypic Variation among European Arctic and Alpine Populations of Oxyria digyna.

*Arctic, Antarctic, and Alpine Research* **37**: 233–238.

**Heide OM, Gauslaa Y**. **1999**. Developmental Strategies of Koenigia Islandica, a High-Arctic Annual Plant. *Ecography* **22**: 637–642.

**Heide OM, Pedersen K, Dahl E**. **1990**. Environmental control of flowering and morphology in the high- arctic Cerastium regelii, and the taxonomic status of C. jenisejense. *Nordic Journal of Botany* **10**:

141–147.

**Heinze W, Midasch M**. **1991**. PHOTOPERIODIC REACTION OF PHYSALIS-PERUVIANA.

*Gartenbauwissenschaft* **56**: 262–264.

**Heller A, Borochov A, Halevy AH**. **1994**. Factors affecting rooting ability of Coleonema aspalathoides.

*Scientia Horticulturae* **58**: 335–341.

**Heslop-Harrison J**. **1960**. Suppressive Effects of 2-Thiouracil on Differentiation and Flowering in Cannabis sativa. *Science* **132**: 1943–1944.

**Hess D**. **1959**. DIE SELEKTIVE BLOCKIERUNG EINES AN DER BLÜHINDUKTION

BETEILIGTEN RIBOSENUCLEINSÄURE-EIWEISS-SYSTEMS DURCH 2-THIOURACIL (UNTERSUCHUNGEN AN STREPTOCARPUS WENDLANDII). *Planta* **54**: 74–94.

**Hettasch HB, Jacobs G**. **2006**. LEUCADENDRONS ARE SHORT-DAY PLANTS: A PRELIMINARY

REPORT. *Acta Horticulturae*: 113–116.

**Im NH, Lim SH, Lee HB, An SK, Lee SY, Kim KS**. **2020**. Growth and flowering responses of Lysimachia mauritiana Lam. to cold treatment and photoperiod. *Scientia Horticulturae* **270**: 109429.

**ISON R, PARSON A**. **1992**. COMPARATIVE GROWTH AND DEVELOPMENT OF KENYA CLOVER (TRIFOLIUM-SEMIPILOSUM) AND WHITE CLOVER (TRIFOLIUM-REPENS CV HAIFA) .2. TEMPERATURE AND DAYLENGTH EFFECTS ON FLOWERING. *Tropical Grasslands* **26**: 51–57.

**Kanaya T, Kokubun H, Watanabe H, Hashimoto G, Marchesi E, Bullrich L, Ando T**. **2010**. Flowering ability of commercial Calibrachoa cultivars as compared to that of natural species. *Scientia Horticulturae* **126**: 276–283.

**Kefu Z, Hai F, Xingyu J, San Z**. **2002**. Critical day-length and photoinductive cycles for the induction of flowering in halophyte Suaeda salsa. *Plant Science* **162**: 27–31.

**Keller F, Korner C**. **2003**. The Role of Photoperiodism in Alpine Plant Development. *Arctic, Antarctic, and Alpine Research* **35**: 361–368.

**Kinet JM**. **1972**. Sinapis alba, a Plant requiring a Single Long Day or a Single Short Day for Flowering.

*Nature* **236**: 406–407.

**King RW**. **1998**. Dual Control of Flower Initiation and Development by Temperature and Photoperiod in Hardenbergia violacea. *Australian Journal of Botany* **46**: 65–74.

**King RW, Worrall R, Dawson IA**. **2008**. Diversity in environmental controls of flowering in Australian plants. *Scientia Horticulturae* **118**: 161–167.

**Kooyers NJ, James B, Blackman BK**. **2017**. Competition drives trait evolution and character displacement between Mimulus species along an environmental gradient. *Evolution* **71**: 1205–1221.

**Krisantini, Wickramasinghe P, Wickramasinghe V, Johnston M**. **2015**. Effect of photoperiod and temperature on flowering of Rhodanthe floribunda and Pycnosorus thompsonianus. *New Zealand Journal of Crop and Horticultural Science* **43**: 275–281.

**Lane HC, Cathey HM, Evans LT**. **1965**. The Dependence of Flowering in Several Long-Day Plants on the Spectral Composition of Light Extending the Photoperiod. *American Journal of Botany* **52**:

1006–1014.

**Laurie A, Poesch GH**. **1932**. Photoperiodism: the value of supplementary illumination and reduction of light on flowering plants in the greenhouse.

**Mattson NS, Erwin JE**. **2005**. The impact of photoperiod and irradiance on flowering of several herbaceous ornamentals. *Scientia Horticulturae* **104**: 275–292.

**Meijer G, Van der Veen R**. **1957**. Wavelength dependence on photoperiodic responses. *Acta botanica*

*neerlandica* **6**: 429–433.

**Michniewicz M, Kamieńska A**. **1965**. Flower formation induced by kinetin and vitamin E treatment in long-day plant (Arabidopsis thaliana) grown in short day. *Naturwissenschaften* **52**: 623–623.

**Millhollon RW, Burner DM**. **1993**. Itchgrass (Rottboellia cochinchinensis) Biotypes in World Populations. *Weed Science* **41**: 379–387.

**Moe R**. **1990**. Effect of day and night temperature alternations and of plant growth regulators on stem elongation and flowering of the long-day plant Campanula isophylla Moretti. *Scientia Horticulturae* **43**: 291–305.

**Novy A, Flory S l., Hartman JM**. **2013**. Evidence for rapid evolution of phenology in an invasive grass.

*Journal of Evolutionary Biology* **26**: 443–450.

**Parker MW, Hendricks SB, Borthwick HA**. **1950**. Action Spectrum for the Photoperiodic Control of Floral Initiation of the Long-Day Plant Hyoscyamus niger. *Botanical Gazette* **111**: 242–252.

**Parker MW, Hendricks SB, Borthwick HA, Scully NJ**. **1946**. Action Spectrum for the Photoperiodic Control of Floral Initiation of Short-Day Plants. *Botanical Gazette* **108**: 1–26.

**Peterson ML, Loomis WE**. **1949**. Effects of Photoperiod and Temperature on Growth and Flowering of Kentucky Bluegrass1. *Plant Physiology* **24**: 31–43.

**REZANSOFF D, HUNER N**. **1993**. THE EFFECTS OF LIGHT, TEMPERATURE AND PHOTOPERIOD ON THE PHOTOINHIBITORY RESPONSE OF PERIWINKLE (VINCA-MINOR L). In: AMER SOC PLANT PHYSIOLOGISTS 15501 MONONA DRIVE, ROCKVILLE, MD 20855, 138–138.

**Runkle ES, Heins RD, Cameron AC, Carlson WH**. **1998**. Flowering of Herbaceous Perennials under Various Night Interruption and Cyclic Lighting Treatments. *HortScience HortSci* **33**: 672–677.

**Runkle ES, Heins RD, Cameron AC, Carlson WH**. **1999**. Cold treatment modifies the photoperiodic flowering response of Lobelia×speciosa. *Scientia Horticulturae* **80**: 247–258.

**Sachs RM**. **1956**. Floral Initiation in Cestrum nocturnum, A Long-Short Day Plant. II. A 24-Hour Versus a 16-Hour Photoperiod for Long Day Induction. *Plant Physiology and Biochemistry* **31**: 429.

**Salisbury FB**. **1981**. Twilight Effect: Initiating Dark Measurement in Photoperiodism of Xanthium. *Plant Physiology* **67**: 1230–1238.

**Scalone R, Lemke A, Štefanić E, Kolseth A-K, Rašić S, Andersson L**. **2016**. Phenological Variation in Ambrosia artemisiifolia L. Facilitates Near Future Establishment at Northern Latitudes. *PLOS ONE* **11**: e0166510.

**Sceglova OA, Leisle FF**. **1954**. *Doklady Akademii Nauk* **95**: 893–95.

**Schuster M, Kandeler R**. **1970**. SIGNIFICANCE OF PHOTOSYNTHESIS FOR LONG-DAY FLOWERING OF SHORT-DAY PLANT LEMNA-PERPUSILLA-6746. *Zeitschrift Fur*

*Pflanzenphysiologie* **63**: 308-.

**SCHWABE WW**. **1959**. Studies of Long-day Inhibition in Short-day Plants. *Journal of Experimental Botany* **10**: 317–329.

**Shen P, Gao S, Hu J, Li Y, Lei T, Shi L**. **2021**. In vitro flowering of the distylous plant Plumbago auriculata Lam. *South African Journal of Botany* **137**: 492–498.

**Shillo R, Weiner A, Halevy AH**. **1985**. Environmental and chemical control of growth and flowering of Chamelaucium uncinatum Schauer. *Scientia Horticulturae* **25**: 287–297.

**Snyder WE**. **1948**. Mechanism of the Photoperiodic Response of Plantago lanceolata L., A Long-Day Plant. *American Journal of Botany* **35**: 520–525.

**Takeda T**. **1996**. The Differences in the Effects of Low Temperature and Day Length on the Flowering within Seed-Propagated *Dianthus* spp. and Cultivars. *Journal of the Japanese Society for Horticultural Science* **65**: 615–623.

**Takeno K, Watanabe K, Suyama T**. **1995**. Sex Determination of Flowers of Salsola komarovii Iljin by Photoperiod. *Journal of Plant Physiology* **146**: 672–676.

**TeltscherovÁ L, SeidlovÁ F, Krekule J**. **1967**. Effect of some pyrimidine analogues on flowering of long-day and short-day plants. *Biologia Plantarum* **9**: 234.

**Thomas RG**. **1961**. Flower Initiation in Trifolium repens L.: a Short-Long-Day Plant. *Nature* **190**: 1130–1131.

**Thomas B, Vince-Prue D**. **1997**. Appendix I - Photoperiodic Classification of Plants. In: Vince-Prue D, ed. Photoperiodism in Plants (Second Edition). London: Academic Press, 355–365.

**Trongkongsin K, Humphreys LR**. **1988**. The long-short day requirement for flowering in Stylosanthes guianensis. *Australian Journal of Agricultural Research* **39**: 199–207.

**Venkataraman R, Seth PN, Maheshwari SC**. **1970**. STUDIES ON GROWTH AND FLOWERING OF A SHORT-DAY PLANT, WOLFFIA-MICROSCOPICA .1. GENERAL ASPECTS AND

INDUCTIONOF FLOWERING BY CYTOKININS. *Zeitschrift Fur Pflanzenphysiologie* **62**: 316.

**Walck JL, Baskin JM, Baskin CC**. **1999**. Ecology of the Endangered Species Solidago shortii. VII. Survivorship and Flowering, and Comparison with Common, Geographically-Widespread Solidago Species. *The Journal of the Torrey Botanical Society* **126**: 124–132.

**Wang X**. **2007**. Gender-specific flowering responses to day length in the dioecious plant Silene latifolia at different temperatures. *Sexual Plant Reproduction* **20**: 45–50.

**Wareing PF**. **1950**. Growth Studies in Woody Species I. Photoperiodism in First-Year Seedlings of Pinus silvestris. *Physiologia Plantarum* **3**: 258–276.

**Wareing PF**. **1956**. Photoperiodism in Woody Plants. *Annual Review of Plant Physiology* **7**: 191–214.

**Wareing P, Longman K**. **1960**. Studies on the physiology of flowering in forest trees. *Studies on the physiology of flowering in forest trees.* **Report on Forest Research by the Forestry Commission for the year ended March, 1959**: 109–10.

**Warner RM, Erwin JE**. **2001**. Variation in floral induction requirements of Hibiscus sp. *Journal of the American Society for Horticultural Science* **126**: 262–268.

**Wellensiek SJ**. **1985**. Campanula medium. In: Halevy AH, ed. Handbook of Flowering. Florida: C.R.C. Press, 123–126.

**Whitton B, Healy W, Roh M**. **1991**. Flowering of Aeschynanthus `Koral’. *HortScience* **26**: 858–859.

**Wisskirchen R**. **2006**. An experimental study on the growth and flowering of riparian pioneer plants under long- and short-day conditions. *Flora - Morphology, Distribution, Functional Ecology of Plants* **201**: 3–23.

**Withrow R, Benedict H**. **1936**. Photoperiodic responses of certain greenhouse annuals as influenced by intensity and wavelength of artificial light used to lengthen the daylight period. *Plant physiology* **11**: 225.

**Withrow RB, Biebel JP**. **1936**. Photoperiodic response of certain long and short day plants to filtered radiation applied as supplement to daylight. *Plant Physiology* **11**: 807–819.

**Wu C, Owen MDK**. **2014**. When Is the Best Time to Emerge: Reproductive Phenology and Success of Natural Common Waterhemp (Amaranthus rudis) Cohorts in the Midwest United States? *Weed Science* **62**: 107–117.

**Younis AF**. **1955**. Studies on the Photoperiodism of Kalanchoë Blossfeldiana. *Physiologia Plantarum* **8**: 223–229.

**Zaccai M, Edri N**. **2002**. Floral transition in lisianthus (Eustoma grandiflorum). *Scientia Horticulturae*

**95**: 333–340.

**Zaidan LBP, Dietrich SMC, Schwabe WW**. **1991**. Effects of temperature and photoperiod on flowering in Hyptis brevipes. *Physiologia Plantarum* **81**: 221–226.

**Supplementary methods 4:** README text and descriptions for supplementary tables.

Appendix 4a. Description of data in supplementary table 1: Photoperiod data

This csv contains information on the photoperiod sensitive flowering requirements of various plant species gathered from published literature for manuscript titled "Species that require long day conditions to flower are not advancing their flowering phenology as fast as species without photoperiod requirements."

Appendix Table 2. Description of data columns in Photoperiod data table.

| Column | Description |
| --- | --- |
| species | Species name in the format of *Genus species* after spelling had been checked.  Cultivars, subspecies and duplicate species that were originally recorded as different species before name check were recorded in separate columns but can be resolved using order column. |
| photoperiod | Photoperiod sensitivity requirements of the plant according to the reference source.  ad = ambiphotoperiodic (requires either short or long daylengths)  id = intermediate (requires daylengths of around 12 hours)  ld = long day (requires daylengths greater than 12 hours)  lsd = long-short day (requires long days that are shortening)  na = no daylength requirement  sd = short day (requires daylengths of shorter than 12 hours)  sld = short-long day (requires short days that are lengthening)  yes = daylength requirement exists but has not yet been confirmed |
| cultivar | The cultivar of the species tested; or common names that the plant is also known under. |
| reference | Reference in which the photoperiod data is found.  Please see Supplementary methods 4 for exact citation. |
| notes | Additional notes |
| sensitivity_yn | Whether there is a photoperiod sensitivity requirement or not. |
| order | The order in which the record was considered in case of multiple photoperiod requirements reported in the literature. One denotes a top priority source.  When records conflicted, we chose experiments that tested more day length intervals, were conducted more recently and tested on wild type individuals. |

Appendix Table 3: Proportion of species in dataset based on photoperiod sensitive flowering requirements.

| Photoperiod sensitive flowering requirement | Number of species |
| --- | --- |
| Short day | 209 |
| Long day | 296 |
| Day neutral | 191 |
| Other | 45 |
| Total | **741** |


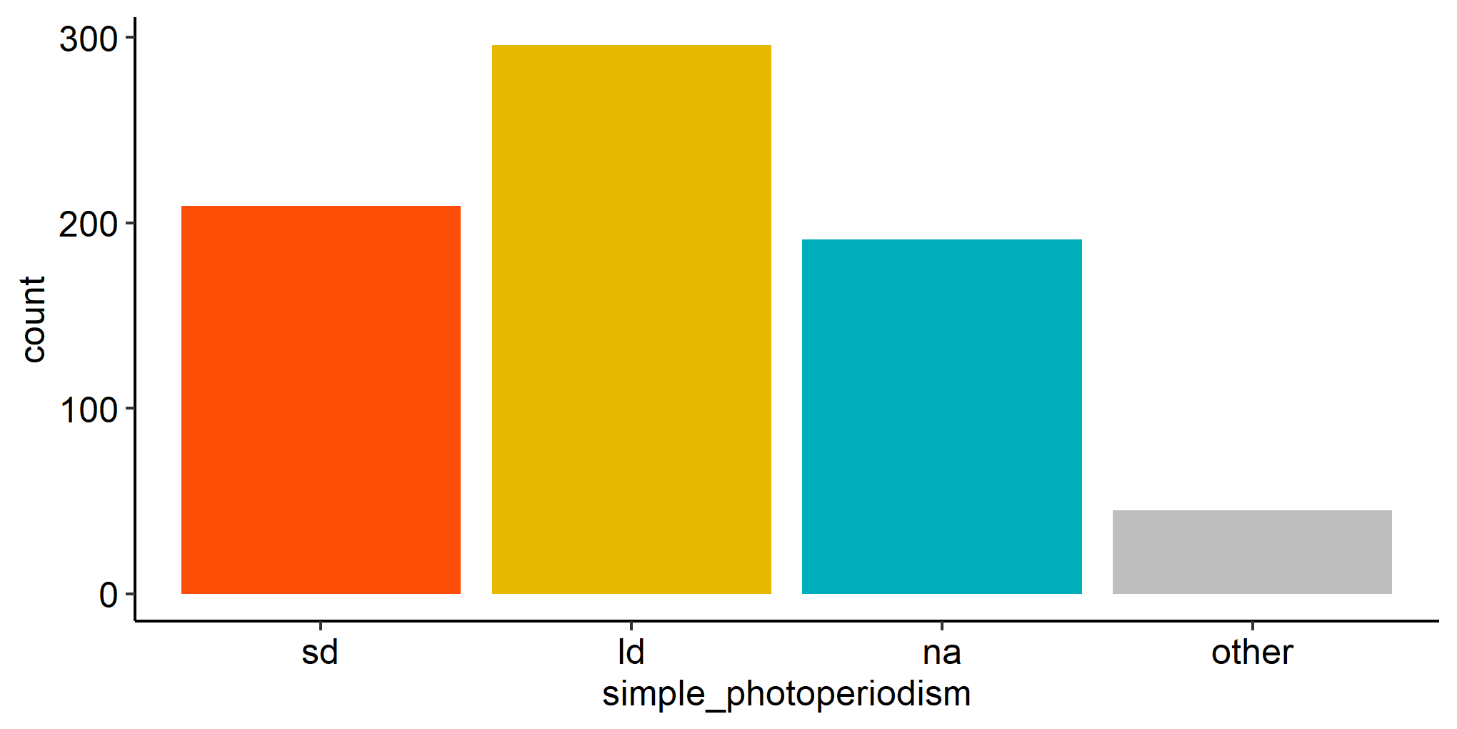


Appendix Figure 1: Proportion of species in dataset based on photoperiod sensitive flowering requirements.

Appendix Table 4: Summary of plant families and genera represented in the flowering time dataset. Note that we have only included families represented by more than 5 species, plus Orchidaceae which was particularly poorly represented..

| Family | n genus | n species | Proportion of dataset (%) |
| --- | --- | --- | --- |
| POACEAE | 46 | 98 | 13.22 |
| ASTERACEAE | 51 | 92 | 12.42 |
| FABACEAE | 25 | 46 | 6.21 |
| SOLANACEAE | 13 | 33 | 4.46 |
| CARYOPHYLLACEAE | 11 | 30 | 4.05 |
| MALVACEAE | 7 | 30 | 4.05 |
| AMARANTHACEAE | 16 | 26 | 3.51 |
| BRASSICACEAE | 14 | 26 | 3.51 |
| ROSACEAE | 11 | 19 | 2.56 |
| CRASSULACEAE | 5 | 17 | 2.29 |
| LAMIACEAE | 11 | 17 | 2.29 |
| ARACEAE | 5 | 14 | 1.89 |
| CONVOLVULACEAE | 3 | 12 | 1.62 |
| CYPERACEAE | 2 | 12 | 1.62 |
| AMARYLLIDACEAE | 4 | 11 | 1.48 |
| POLYGONACEAE | 6 | 11 | 1.48 |
| RANUNCULACEAE | 6 | 11 | 1.48 |
| CAMPANULACEAE | 4 | 10 | 1.35 |
| APIACEAE | 9 | 9 | 1.21 |
| ONAGRACEAE | 2 | 9 | 1.21 |
| PRIMULACEAE | 5 | 9 | 1.21 |
| LILIACEAE | 2 | 8 | 1.08 |
| PLANTAGINACEAE | 5 | 8 | 1.08 |
| ASPARAGACEAE | 4 | 6 | 0.81 |
| CUCURBITACEAE | 4 | 6 | 0.81 |
| EUPHORBIACEAE | 3 | 6 | 0.81 |
| POLEMONIACEAE | 5 | 6 | 0.81 |
| RUBIACEAE | 6 | 6 | 0.81 |
| SCROPHULARIACEAE | 2 | 6 | 0.81 |
| ACANTHACEAE | 4 | 5 | 0.67 |
| APOCYNACEAE | 5 | 5 | 0.67 |
| BORAGINACEAE | 4 | 5 | 0.67 |
| ERICACEAE | 3 | 5 | 0.67 |
| IRIDACEAE | 4 | 5 | 0.67 |
| PAPAVERACEAE | 4 | 5 | 0.67 |
| SAXIFRAGACEAE | 1 | 5 | 0.67 |
| ORCHIDACEAE | 2 | 2 | 0.27 |

Appendix 4b. Description of data in supplementary table 2 : First flowering day data

This csv contains information on the historic first flowering days for each species for manuscript titled "Species that require long day conditions to flower are not advancing their flowering phenology as fast as species without photoperiod requirements."

Please contact NaturesCalendar@woodlandtrust.org.uk for further information regarding Nature's Calendar data and/or the wider phenology dataset, which is freely available to students and researchers (T&Cs apply).

Appendix Table 5: Description of data tables used in First flowering day data table.

| Column | Description |
| --- | --- |
| species | Species name in the format of *Genus species*. |
| year | Year in which record was observed. |
| doy | Day of year in which first flowering was observed. |
| source | Published source of the record.  uknc = UK Nature’s Calendar  fitter = Fitter & Fitter (2002)  abuasab = Abu-Asab *et al.*(2001)  rmbl = Rocky Mountain Biological Laboratory Phenology Project  park = Park *et al.* (2019)  pep725 = Pan European Phenology Project  uspn = USA National Phenology Network |
| location | Name of location that was used as a basis for centroid coordinates if none were otherwise provided in source. |
| lat | Latitude in WGS84. |
| lon | Longitude in WGS84. |

**Methods S5** Summary of model outputs

Below are descriptions of each model used in our main analysis, as well as the fixed effects outputs for each model. For a complete output please see the associated github project.

Appendix Table 6: Comparison of models and their parameters

| Model |
| --- |
| Model 1: Linear Model  First flowering day ~ year  + latitude  + (1\|species) |
| Model 2: Linear Model with photoperiod  First flowering day ~ year*photoperiod  + latitude:photoperiod  + (1\|species) |
| Model 3: Phylogenetic ANOVA  year_effect_vector = slope of year in:  First flowering day ~ year + latitude  phylANOVA(tree, x = simple_photoperiodism_vector, y = year_effect_vector, nsim = 9999, posthoc = TRUE) |
| Analysis 4: Type 2 ANOVA of Model 1  Car::Anova(Model 1) |
| Analysis 5: Estimated Marginal trends for each photoperiod group and subsequent contrasts between the groups |

Appendix Table 7: Model 1: The relationship between flowering time and year. Species is included as a random effect.

|  | Estimate | SE | df | t-value | p-value |
| --- | --- | --- | --- | --- | --- |
| Intercept | 6.756e+01 | 2.335e+01 | 1.599e+04 | 2.894 | 0.00381* |
| year | 2.455e-02 | 1.193e-02 | 2.161e+04 | 2.058 | 0.03962* |
| latitude | 7.752e-01 | 3.529e-02 | 2.161e+04 | 21.970 | < 0.0001* |

Appendix Table 8: Model 2: The relationship between flowering time and year, and the effect that photoperiod sensitivity and latitude have on the relationships.

|  | Estimate | SE | df | t-value | p-value |
| --- | --- | --- | --- | --- | --- |
| Intercept | 268.7 | 35.92 | 13750 | 7.479 | < 0.0001 * |
| ld | -207.3 | 52.08 | 15790 | -3.981 | < 0.0001* |
| sd | 345.7 | 98.75 | 20790 | 3.501 | < 0.001* |
| year (na:year) | -0.094 | 0.018 | 21570 | -5.077 | < 0.0001* |
| ld:year | 0.11 | 0.026 | 21570 | 4.276 | < 0.0001* |
| Sd:year | -0.04.83 | 0.048 | 21600 | -1.005 | 0.31 |
| na:latitude | 1.119 | 0.049 | 21580 | 22.834 | < 0.0001* |
| ld:latitude | 1.119 | 0.075 | 21600 | 15.015 | < 0.0001* |
| sd:latitude | -3.654 | 0.17 | 21210 | -20.970 | < 0.0001* |

Appendix Table 9: Model 3: Whether there was a significant difference in the relationship between flowering time and year based on photoperiod sensitivity, after effects of latitude and phylogeny are taken into account. Individual linear models for each species of the first flowering day over time included latitude as a fixed effect. Pairwise corrected p-values are based on simulation (nsim = 9999, method = holm).

|  | ld | na | sd |
| --- | --- | --- | --- |
| ld | 1 | 0.787279 | 0.038704 |
| na | 0.787279 | 1 | 0.094609 |
| sd | 0.038704 | 0.094609 | 1 |

Appendix Table 10: Analysis of Deviance (Type II Wald chisquare test) for Model 2

|  | chisq | df | p-val |
| --- | --- | --- | --- |
| Photoperiodism | 7.49 | 2 | 0.02* |
| Year | 13.68 | 1 | > 0.001* |
| Photoperiodism:year | 23.40 | 2 | > 0.0001* |
| Photoperiodism:Latitude | 1186.58 | 3 | > 0.0001* |

Appendix Table 11: Comparison of Model 1 and Model 2 to confirm that the addition of photoperiod information improved fit when modelling changes in first flowering day.

|  | AIC | BIC | log-  likelihood | deviance | chisq | df | p-val |
| --- | --- | --- | --- | --- | --- | --- | --- |
| Model 1  (without photoperiod) | 193314 | 193347 | -96649 | 193297 | na | na | na |
| Model 2  (with photoperiod) | 192564 | 192643 | -96267 | 192533 | 764.24 | 6 | <0.0001 |

Appendix Table 12: Estimated Marginal Trends for Model 2. Note that degrees of freedom exceeded limit and therefore asymptotic degrees of freedom was applied to calculate upper and lower confidence limits.

|  | trend (year) | Se | df | lower CL | upper CL |
| --- | --- | --- | --- | --- | --- |
| Day neutral | -0.94 | 0.019 | Inf | -0.13 | -0.06 |
| Long day | 0.018 | 0.019 | Inf | -0.02 | 0.05 |
| Short day | -0.14 | 0.044 | inf | -0.23 | -0.06 |

Appendix Table 13: Paired contrasts between estimated marginal trends. Note that p value was adjusted for 3 estimates using the tukey method.

|  | estimate | se | df | z | p-val |
| --- | --- | --- | --- | --- | --- |
| Day neutral – long day | -0.112 | 0.026 | Inf | -4.28 | 0.0001 |
| Day neutral – short day | 0.048 | 0.048 | Inf | 1.01 | 0.57 |
| Long day – short day | 0.161 | 0.048 | Inf | 3.34 | < 0.001 |

**Methods S6** Species used in each analysis

1. Photoperiod sensitivity and flowering time

*Achillea millefolium*

*Agrimonia eupatoria*

*Ambrosia artemisiifolia*

*Anagallis arvensis*

*Antirrhinum majus*

*Aquilegia canadensis*

*Arabidopsis thaliana*

*Arctium lappa*

*Arenaria serpyllifolia*

*Arrhenatherum elatius*

*Asclepias tuberosa*

*Atriplex prostrata*

*Avena sativa*

*Betula pendula*

*Bothriochloa bladhii*

*Brachypodium pinnatum*

*Brassica napus*

*Briza media*

*Bromus inermis*

*Calluna vulgaris*

*Chenopodium album*

*Cichorium intybus*

*Cornus florida*

*Cynodon dactylon*

*Cynosurus cristatus*

*Dactylis glomerata*

*Datura stramonium*

*Daucus carota*

*Deschampsia cespitosa*

*Digitalis purpurea*

*Epilobium parviflorum*

*Fagus sylvatica*

*Festuca arundinacea*

*Festuca pratensis*

*Festuca rubra*

*Fragaria vesca*

*Geum canadense*

*Geum urbanum*

*Helianthus annuus*

*Helianthus tuberosus*

*Hemerocallis fulva*

*Hibiscus moscheutos*

*Hibiscus trionum*

*Holcus lanatus*

*Humulus lupulus*

*Ilex aquifolium*

*Ipomoea hederacea*

*Lactuca serriola*

*Lespedeza cuneata*

*Lolium perenne*

*Lunaria annua*

*Myosotis scorpioides*

*Oenothera biennis*

*Panicum virgatum*

*Papaver rhoeas*

*Papaver somniferum*

*Pastinaca sativa*

*Persicaria lapathifolia*

*Phleum pratense*

*Pinus sylvestris*

*Plantago lanceolata*

*Plantago major*

*Poa nemoralis*

*Poa pratensis*

*Poa trivialis*

*Portulaca oleracea*

*Punica granatum*

*Pyrus communis*

*Ribes nigrum*

*Ribes rubrum*

*Rosa gallica*

*Rubus idaeus*

*Rudbeckia hirta*

*Rumex acetosella*

*Scrophularia vernalis*

*Setaria viridis*

*Silene dioica*

*Silene latifolia*

*Sonchus oleraceus*

*Sorghum halepense*

*Syringa vulgaris*

*Trifolium pratense*

*Ulmus glabra*

*Vaccinium angustifolium*

*Vaccinium corymbosum*

*Vinca minor*

*Viola odorata*

*Xanthium strumarium*

1. Photoperiod sensitivity and flowering time with phylogeny

*Achillea millefolium*

*Agrimonia eupatoria*

*Alopecurus pratensis*

*Anagallis arvensis*

*Antirrhinum majus*

*Aquilegia canadensis*

*Arabidopsis thaliana*

*Arctium lappa*

*Arrhenatherum elatius*

*Atriplex prostrata*

*Avena sativa*

*Betula pendula*

*Brachypodium pinnatum*

*Brassica napus*

*Briza media*

*Calluna vulgaris*

*Chenopodium album*

*Cichorium intybus*

*Cornus florida*

*Cynosurus cristatus*

*Dactylis glomerata*

*Daucus carota*

*Deschampsia cespitosa*

*Digitalis purpurea*

*Epilobium parviflorum*

*Fragaria vesca*

*Geum urbanum*

*Helianthus annuus*

*Hemerocallis fulva*

*Holcus lanatus*

*Ilex aquifolium*

*Lactuca serriola*

*Lunaria annua*

*Myosotis scorpioides*

*Papaver rhoeas*

*Papaver somniferum*

*Pastinaca sativa*

*Persicaria lapathifolia*

*Phleum pratense*

*Pinus sylvestris*

*Plantago lanceolata*

*Plantago major*

*Poa nemoralis*

*Pyrus communis*

*Ribes nigrum*

*Ribes rubrum*

*Rosa gallica*

*Rubus idaeus*

*Rumex acetosella*

*Silene dioica*

*Silene latifolia*

*Sinapis alba*

*Solidago canadensis*

*Sonchus oleraceus*

*Syringa vulgaris*

*Trifolium pratense*

*Trifolium repens*

*Ulmus glabra*

*Vaccinium angustifolium*

*Vaccinium corymbosum*

*Vinca minor*

*Viola odorata*

1. Photoperiod sensitivity and life history

*Achillea millefolium*

*Agrimonia eupatoria*

*Agrostemma githago*

*Agrostis canina*

*Agrostis capillaris*

*Agrostis stolonifera*

*Agrostis tenuis*

*Aira praecox*

*Allium ampeloprasum*

*Allium cepa*

*Allium sativum*

*Allium sphaerocephalon*

*Alopecurus aequalis*

*Amaranthus caudatus*

*Amaranthus graecizans*

*Amaranthus hybridus*

*Amaranthus retroflexus*

*Amaranthus tricolor*

*Ambrosia artemisiifolia*

*Ammi majus*

*Anagallis arvensis*

*Anagallis tenella*

*Andropogon virginicus*

*Androsace alpina*

*Anethum graveolens*

*Anthemis cotula*

*Anthriscus cerefolium*

*Antirrhinum majus*

*Apium graveolens*

*Arabidopsis thaliana*

*Arctium lappa*

*Arenaria serpyllifolia*

*Arrhenatherum elatius*

*Asclepias tuberosa*

*Asperula arvensis*

*Atriplex prostrata*

*Aurinia saxatilis*

*Avena sativa*

*Beta vulgaris*

*Betula pendula*

*Bidens cernua*

*Bidens radiata*

*Bidens tripartita*

*Blitum capitatum*

*Bouteloua eriopoda*

*Brachypodium pinnatum*

*Brachypodium sylvaticum*

*Brassica juncea*

*Brassica napus*

*Brassica nigra*

*Briza media*

*Bromus erectus*

*Bromus inermis*

*Bromus racemosus*

*Bromus rubens*

*Bromus secalinus*

*Calendula officinalis*

*Calluna vulgaris*

*Calystegia sepium*

*Campanula alliariifolia*

*Campanula carpatica*

*Campanula medium*

*Campanula persicifolia*

*Cardamine amara*

*Carex canescens*

*Carex curvula*

*Carex echinata*

*Carex lachenalii*

*Carex magellanica*

*Carex maritima*

*Carex paupercula*

*Catananche caerulea*

*Centaurea cyanus*

*Chenopodium album*

*Chenopodium ficifolium*

*Chenopodium glaucum*

*Chenopodium murale*

*Chenopodium polyspermum*

*Chenopodium rubrum*

*Chenopodium vulvaria*

*Chondrilla juncea*

*Chrysanthemum coronarium*

*Chrysanthemum morifolium*

*Cicer arietinum*

*Cichorium intybus*

*Coriandrum sativum*

*Cosmos bipinnatus*

*Cucumis melo*

*Cucumis sativus*

*Cynara scolymus*

*Cynodon dactylon*

*Cynosurus cristatus*

*Dactylis glomerata*

*Datura stramonium*

*Daucus carota*

*Delphinium elatum*

*Deschampsia cespitosa*

*Dianthus arenarius*

*Dianthus barbatus*

*Dianthus carthusianorum*

*Dianthus caryophyllus*

*Dianthus superbus*

*Dichondra repens*

*Digitalis purpurea*

*Draba aizoides*

*Elyna myosuroides*

*Epilobium parviflorum*

*Euphorbia lathyris*

*Fagopyrum esculentum*

*Fagus sylvatica*

*Festuca arundinacea*

*Festuca gigantea*

*Festuca ovina*

*Festuca pratensis*

*Festuca rubra*

*Foeniculum vulgare*

*Fragaria vesca*

*Fragaria x ananassa*

*Gardenia jasminoides*

*Gentiana bavarica*

*Geum reptans*

*Geum urbanum*

*Glycine max*

*Gnaphalium supinum*

*Gossypium hirsutum*

*Gypsophila elegans*

*Gypsophila paniculata*

*Gypsophila repens*

*Helianthus annuus*

*Helianthus tuberosus*

*Hemerocallis fulva*

*Hibiscus trionum*

*Hieracium floribundum*

*Holcus lanatus*

*Hordeum marinum*

*Hordeum vulgare*

*Humulus lupulus*

*Hyacinthus orientalis*

*Hyoscyamus niger*

*Iberis umbellata*

*Ilex aquifolium*

*Impatiens balsamina*

*Koenigia islandica*

*Lactuca sativa*

*Lactuca serriola*

*Lagurus ovatus*

*Lathyrus odoratus*

*Legousia speculum-veneris*

*Lemna gibba*

*Lemna minor*

*Leucanthemopsis alpina*

*Linaria alpina*

*Linum perenne*

*Linum usitatissimum*

*Lolium perenne*

*Lolium temulentum*

*Lunaria annua*

*Lupinus luteus*

*Luzula spicata*

*Lycopersicon esculentum*

*Lysimachia nemorum*

*Malus sylvestris*

*Malva verticillata*

*Matthiola incana*

*Medicago minima*

*Medicago sativa*

*Melilotus alba*

*Mentha spicata*

*Mimulus guttatus*

*Minuartia sedoides*

*Muscari botryoides*

*Myosotis scorpioides*

*Narcissus tazetta*

*Nicotiana tabacum*

*Nigella damascena*

*Oenothera biennis*

*Oenothera parviflora*

*Oenothera rosea*

*Oryza sativa*

*Oxalis pes-caprae*

*Oxyria digyna*

*Panicum maximum*

*Panicum miliaceum*

*Panicum virgatum*

*Papaver rhoeas*

*Papaver somniferum*

*Paspalum urvillei*

*Pastinaca sativa*

*Persicaria lapathifolia*

*Phacelia tanacetifolia*

*Phalaris arundinacea*

*Phalaris canariensis*

*Phalaris minor*

*Phaseolus lunatus*

*Phaseolus vulgaris*

*Phleum pratense*

*Phlox paniculata*

*Physalis peruviana*

*Pinus sylvestris*

*Pisum sativum*

*Plantago lanceolata*

*Plantago major*

*Poa alpina*

*Poa bulbosa*

*Poa laxa*

*Poa nemoralis*

*Poa palustris*

*Poa pratensis*

*Poa trivialis*

*Polypogon monspeliensis*

*Portulaca oleracea*

*Pyrus communis*

*Ranunculus glacialis*

*Ranunculus penicillatus*

*Ranunculus sceleratus*

*Rhodanthe floribunda*

*Ribes nigrum*

*Ribes rubrum*

*Ricinus communis*

*Rosa gallica*

*Rosa rugosa*

*Rubus idaeus*

*Rudbeckia hirta*

*Rumex acetosella*

*Rumex palustris*

*Saxifraga bryoides*

*Saxifraga hypnoides*

*Saxifraga oppositifolia*

*Saxifraga rotundifolia*

*Saxifraga seguieri*

*Scabiosa atropurpurea*

*Scabiosa canescens*

*Scrophularia peregrina*

*Scrophularia umbrosa*

*Scrophularia vernalis*

*Secale cereale*

*Sedum alpestre*

*Sedum spurium*

*Sedum telephium*

*Senecio vulgaris*

*Sesamum indicum*

*Setaria italica*

*Setaria viridis*

*Silene coeli-rosa*

*Silene dioica*

*Silene italica*

*Silene latifolia*

*Silene nutans*

*Silene otites*

*Solanum melongena*

*Solanum tuberosum*

*Sonchus oleraceus*

*Sorghum bicolor*

*Sorghum halepense*

*Suaeda salsa*

*Syringa vulgaris*

*Teucrium scorodonia*

*Thlaspi perfoliatum*

*Thuja plicata*

*Trifolium pratense*

*Trifolium subterraneum*

*Triticum aestivum*

*Tulipa gesneriana*

*Ulmus glabra*

*Verbascum phoeniceum*

*Vicia faba*

*Vigna unguiculata*

*Vinca minor*

*Viola odorata*

*Viola tricolor*

*Vitis vinifera*

*Xanthium strumarium*

*Zea mays*

1. Photoperiod sensitivity and growth form

*Achillea millefolium*

*Aechmea fasciata*

*Agrimonia eupatoria*

*Agrostemma githago*

*Agrostis alba*

*Agrostis canina*

*Agrostis capillaris*

*Agrostis stolonifera*

*Agrostis tenuis*

*Aira praecox*

*Allium ampeloprasum*

*Allium cepa*

*Allium sphaerocephalon*

*Allium tuberosum*

*Alopecurus aequalis*

*Amaranthus caudatus*

*Amaranthus graecizans*

*Amaranthus hybridus*

*Amaranthus retroflexus*

*Amaranthus tricolor*

*Ambrosia artemisiifolia*

*Ammi majus*

*Anagallis arvensis*

*Anagallis tenella*

*Ananas comosus*

*Andropogon virginicus*

*Androsace alpina*

*Anethum graveolens*

*Anigozanthos flavidus*

*Anigozanthos pulcherrimus*

*Anigozanthos rufus*

*Anthemis cotula*

*Anthriscus cerefolium*

*Antirrhinum majus*

*Apium graveolens*

*Aquilegia canadensis*

*Arabidopsis thaliana*

*Arachis hypogaea*

*Arctium lappa*

*Arenaria serpyllifolia*

*Arrhenatherum elatius*

*Asclepias tuberosa*

*Asparagus plumosus*

*Asperula arvensis*

*Atriplex prostrata*

*Avena sativa*

*Beta vulgaris*

*Betula pendula*

*Bidens cernua*

*Bidens tripartita*

*Bothriochloa bladhii*

*Bougainvillea glabra*

*Brachypodium pinnatum*

*Brachypodium sylvaticum*

*Brassavola nodosa*

*Brassica campestris*

*Brassica carinata*

*Brassica juncea*

*Brassica napus*

*Brassica nigra*

*Brassica pekinensis*

*Briza media*

*Bromus erectus*

*Bromus inermis*

*Bromus racemosus*

*Bromus rubens*

*Bromus secalinus*

*Browallia speciosa*

*Brunfelsia pauciflora*

*Cajanus cajan*

*Calendula officinalis*

*Calluna vulgaris*

*Calystegia sepium*

*Calytrix fraseri*

*Camellia japonica*

*Campanula alliariifolia*

*Campanula carpatica*

*Campanula medium*

*Cardamine amara*

*Carex canescens*

*Carex curvula*

*Carex echinata*

*Carex lachenalii*

*Carex magellanica*

*Carex maritima*

*Carex paupercula*

*Carica papaya*

*Carpanthea pomeridiana*

*Caryopteris incana*

*Catananche caerulea*

*Catharanthus roseus*

*Centaurea cyanus*

*Cestrum diurnum*

*Chamaecyparis obtusa*

*Chamelaucium uncinatum*

*Chenopodium album*

*Chenopodium ficifolium*

*Chenopodium glaucum*

*Chenopodium murale*

*Chenopodium polyspermum*

*Chenopodium rubrum*

*Chondrilla juncea*

*Chrysanthemum coccineum*

*Chrysanthemum coronarium*

*Chrysanthemum indicum*

*Chrysanthemum maximum*

*Chrysanthemum morifolium*

*Cicer arietinum*

*Cichorium intybus*

*Cleome hassleriana*

*Cobaea scandens*

*Coffea arabica*

*Colchicum tunicatum*

*Collinsia heterophylla*

*Corchorus capsularis*

*Corchorus olitorius*

*Coreopsis grandiflora*

*Coreopsis lanceolata*

*Coreopsis tinctoria*

*Coriandrum sativum*

*Cornus florida*

*Cosmos bipinnatus*

*Cosmos sulphureus*

*Cryptomeria japonica*

*Cucumis melo*

*Cucumis sativus*

*Cupressus arizonica*

*Cyclamen persicum*

*Cynara scolymus*

*Cynodon dactylon*

*Cynosurus cristatus*

*Dactylis glomerata*

*Dalea foliosa*

*Datura stramonium*

*Daucus carota*

*Delphinium elatum*

*Deschampsia cespitosa*

*Desmodium tortuosum*

*Dianthus arenarius*

*Dianthus barbatus*

*Dianthus carthusianorum*

*Dianthus caryophyllus*

*Dianthus superbus*

*Dichondra repens*

*Digitalis purpurea*

*Dimorphotheca sinuata*

*Draba aizoides*

*Epilobium parviflorum*

*Eschscholzia californica*

*Eucharis grandiflora*

*Euphorbia fulgens*

*Euphorbia heterophylla*

*Euphorbia lathyris*

*Euphorbia pulcherrima*

*Fagopyrum esculentum*

*Fagus sylvatica*

*Festuca arundinacea*

*Festuca gigantea*

*Festuca ovina*

*Festuca pratensis*

*Festuca rubra*

*Foeniculum vulgare*

*Fragaria vesca*

*Gaillardia grandiflora*

*Gardenia jasminoides*

*Gentiana bavarica*

*Geum canadense*

*Geum macrophyllum*

*Geum reptans*

*Geum urbanum*

*Gladiolus grandiflorus*

*Glycine max*

*Gnaphalium supinum*

*Gossypium hirsutum*

*Guzmania monostachia*

*Gypsophila paniculata*

*Hamelia patens*

*Hardenbergia violacea*

*Helianthus annuus*

*Helianthus giganteus*

*Helianthus tuberosus*

*Heliconia stricta*

*Heliconia wagneriana*

*Hemerocallis fulva*

*Hibiscus calyphyllus*

*Hibiscus cannabinus*

*Hibiscus costatus*

*Hibiscus engleri*

*Hibiscus laevis*

*Hibiscus meraukensis*

*Hibiscus nigricaulis*

*Hibiscus physaloides*

*Hibiscus rosa-sinensis*

*Hibiscus schinzii*

*Hibiscus surattensis*

*Hibiscus syriacus*

*Hibiscus trionum*

*Hildegardia barteri*

*Holcus lanatus*

*Holcus sudanensis*

*Hordeum marinum*

*Hordeum vulgare*

*Humulus japonicus*

*Humulus lupulus*

*Hyacinthus orientalis*

*Hydrangea macrophylla*

*Hyoscyamus niger*

*Hyparrhenia rufa*

*Iberis umbellata*

*Ilex aquifolium*

*Impatiens balsamina*

*Indigofera hirsuta*

*Ipomoea alba*

*Ipomoea hederacea*

*Ipomoea setosa*

*Iris ensata*

*Iris tingitana*

*Ixia maculata*

*Jasminum grandiflorum*

*Koenigia islandica*

*Lactuca sativa*

*Lactuca serriola*

*Lagenaria siceraria*

*Lagerstroemia indica*

*Lagurus ovatus*

*Lemna aequinoctialis*

*Lemna gibba*

*Lemna minor*

*Lemna paucicostata*

*Lemna perpusilla*

*Lens culinaris*

*Leptospermum scoparium*

*Lespedeza cuneata*

*Leucanthemopsis alpina*

*Lilium candidum*

*Lilium longiflorum*

*Limnanthes douglasii*

*Limonium sinuatum*

*Linaria alpina*

*Linum perenne*

*Lolium perenne*

*Lolium temulentum*

*Lunaria annua*

*Lupinus cosentinii*

*Lupinus luteus*

*Luzula spicata*

*Lycopersicon esculentum*

*Lysimachia mauritiana*

*Lysimachia nemorum*

*Malus sylvestris*

*Malva verticillata*

*Manihot esculenta*

*Matthiola incana*

*Medicago minima*

*Medicago sativa*

*Mentha piperita*

*Mentha spicata*

*Microstegium vimineum*

*Mimulus guttatus*

*Muscari botryoides*

*Myosotis scorpioides*

*Narcissus tazetta*

*Neonotonia wightii*

*Nicotiana sylvestris*

*Nicotiana tabacum*

*Nigella damascena*

*Oenothera biennis*

*Oenothera parviflora*

*Oenothera rosea*

*Olea europaea*

*Oryza sativa*

*Oryzopsis miliacea*

*Osteospermum jucundum*

*Oxyria digyna*

*Panicum maximum*

*Panicum miliaceum*

*Panicum virgatum*

*Papaver rhoeas*

*Papaver somniferum*

*Paspalum thunbergii*

*Paspalum urvillei*

*Pelargonium zonale*

*Pennisetum purpureum*

*Pentas lanceolata*

*Persicaria lapathifolia*

*Phacelia campanularia*

*Phalaris arundinacea*

*Phalaris canariensis*

*Phalaris minor*

*Phaseolus vulgaris*

*Phleum pratense*

*Phlox paniculata*

*Physalis peruviana*

*Pinus sylvestris*

*Pistia stratiotes*

*Pisum sativum*

*Plantago lanceolata*

*Plantago major*

*Plectranthus scutellarioides*

*Plumbago indica*

*Poa alpina*

*Poa bulbosa*

*Poa laxa*

*Poa nemoralis*

*Poa palustris*

*Poa pratensis*

*Poa trivialis*

*Polypogon monspeliensis*

*Portulaca oleracea*

*Primula malacoides*

*Primula obconica*

*Psophocarpus tetragonolobus*

*Punica granatum*

*Pyrus communis*

*Ranunculus glacialis*

*Ranunculus penicillatus*

*Ranunculus sceleratus*

*Rheum rhaponticum*

*Rhodanthe floribunda*

*Rhododendron obtusum*

*Ribes nigrum*

*Ribes rubrum*

*Ricinus communis*

*Rosa gallica*

*Rosa rugosa*

*Rottboellia cochinchinensis*

*Rubus idaeus*

*Rudbeckia hirta*

*Rumex acetosella*

*Rumex palustris*

*Salpiglossis sinuata*

*Salvia occidentalis*

*Saxifraga bryoides*

*Saxifraga oppositifolia*

*Scabiosa atropurpurea*

*Schizanthus pinnatus*

*Schlumbergera truncata*

*Scrophularia peregrina*

*Scrophularia umbrosa*

*Secale cereale*

*Sechium edule*

*Sedum telephium*

*Senecio vulgaris*

*Sesamum indicum*

*Setaria italica*

*Setaria sphacelata*

*Setaria viridis*

*Silene armeria*

*Silene dioica*

*Silene latifolia*

*Silene nutans*

*Silene otites*

*Solanum melongena*

*Solanum tuberosum*

*Sonchus oleraceus*

*Sorghum bicolor*

*Sorghum halepense*

*Spirodela polyrrhiza*

*Spirodela punctata*

*Stephanotis floribunda*

*Stipa barbata*

*Suaeda salsa*

*Syringa vulgaris*

*Tagetes erecta*

*Tagetes patula*

*Tagetes tenuifolia*

*Teucrium scorodonia*

*Thlaspi perfoliatum*

*Thuja plicata*

*Torenia fournieri*

*Trachelium caeruleum*

*Trifolium pratense*

*Trifolium semipilosum*

*Trifolium subterraneum*

*Trigonella arabica*

*Triticum aestivum*

*Tulipa gesneriana*

*Ulmus glabra*

*Vaccinium angustifolium*

*Vaccinium corymbosum*

*Viburnum carlesii*

*Vicia faba*

*Vigna unguiculata*

*Viola odorata*

*Viola papilionacea*

*Viola tricolor*

*Vitis vinifera*

*Vriesea splendens*

*Zantedeschia aethiopica*

*Zea mays*

*Zinnia elegans*

*Zoysia japonica*

*Zoysia matrella*
